# Cardiac Tamponade as the Initial Presentation of Metastatic Esophageal Adenocarcinoma

**DOI:** 10.7759/cureus.16863

**Published:** 2021-08-03

**Authors:** Jose Amadeo Flores Castro, Alaameri Rasha, Asha Pandu, Savi Mushiyev

**Affiliations:** 1 Internal Medicine, Metropolitan Hospital, New York, USA; 2 Internal Medicine, Albany Medical Center, Albany, USA; 3 Cardiology, Metropolitan Hospital Center, New York Medical College, New York, USA

**Keywords:** cardiac tamponade, esophageal adenocarcinoma, chronic pericardial effusion

## Abstract

Malignancy accounts for approximately 15-20% of moderate to large pericardial effusions. Pulmonary and colon are the most common primary causes. Large pleural effusions tend to present with a less dramatic clinical picture. It is because fluids tend to build up slowly, giving enough time to the pericardial sac to accommodate it until pressure reaches a critical value causing right heart chambers to collapse. In this report, we present the case of a 51-year-old male with cardiac tamponade as the first manifestation of esophageal adenocarcinoma. The patient presented with shortness of breath and pleuritic chest pain for one week, with no other associated symptoms. Early workup indicated a cardiac tamponade likely secondary to lung malignancy. Further workup demonstrated that the primary source was an esophageal malignancy. In this setting, pericardial effusions are usually related to radiation/chemotherapy, but in rare cases, cardiac tamponade can be the first manifestation of esophageal cancer.

## Introduction

Cardiac tamponade is a condition where fluids/blood build up in the pericardial sac, thereby producing enough pressure to compress cardiac cavities, subsequently leading to decreased cardiac output. Its severity may vary from chest discomfort and jugular venous distention (JVD) to cardiogenic shock based on the velocity that fluid builds up. Malignancy accounts for approximately 15-20% of moderate to large pericardial effusions [1,2]. Among these, pulmonary cancer (bronchial adenocarcinoma) is the most common one [3]. Non-specific symptoms are the most common initial presentation of this cancer. However, in rare cases, cardiac tamponade is the first symptom to appear.

## Case presentation

A 51-year-old Hispanic male with a past medical history of gastritis, alcohol abuse, amebic liver abscess drainage 30 years ago, who was an active smoker (25 pack-year), presented to the emergency department (ED) complaining of progressive shortness of breath of one week's duration. His current condition was associated with productive cough, white phlegm, pleuritic chest pain, and burning epigastric pain. Symptoms tended to worsen with exertion and alleviated with rest. The patient also complained of sore throat, mild subjective continuous fever, and chills one week ago, which lasted for three days. He also reported an unintentional weight loss of 4 kg during the past five months. He denied palpitations, dizziness, abdominal pain, gastrointestinal (GI) bleeding, recent travel, or any contact with ill people.

The patient was alert, oriented in the three spheres, with a blood pressure of 109/56 mmHg, heart rate of 97 bpm, respiratory rate of 24 per minute, O_2_ saturation of 96%, afebrile, and appeared comfortable on arrival to ED. The physical exam was remarkable for JVD, decreased breath sounds on the right side of the thorax, and abdominal distension without tenderness.

**Figure 1:**
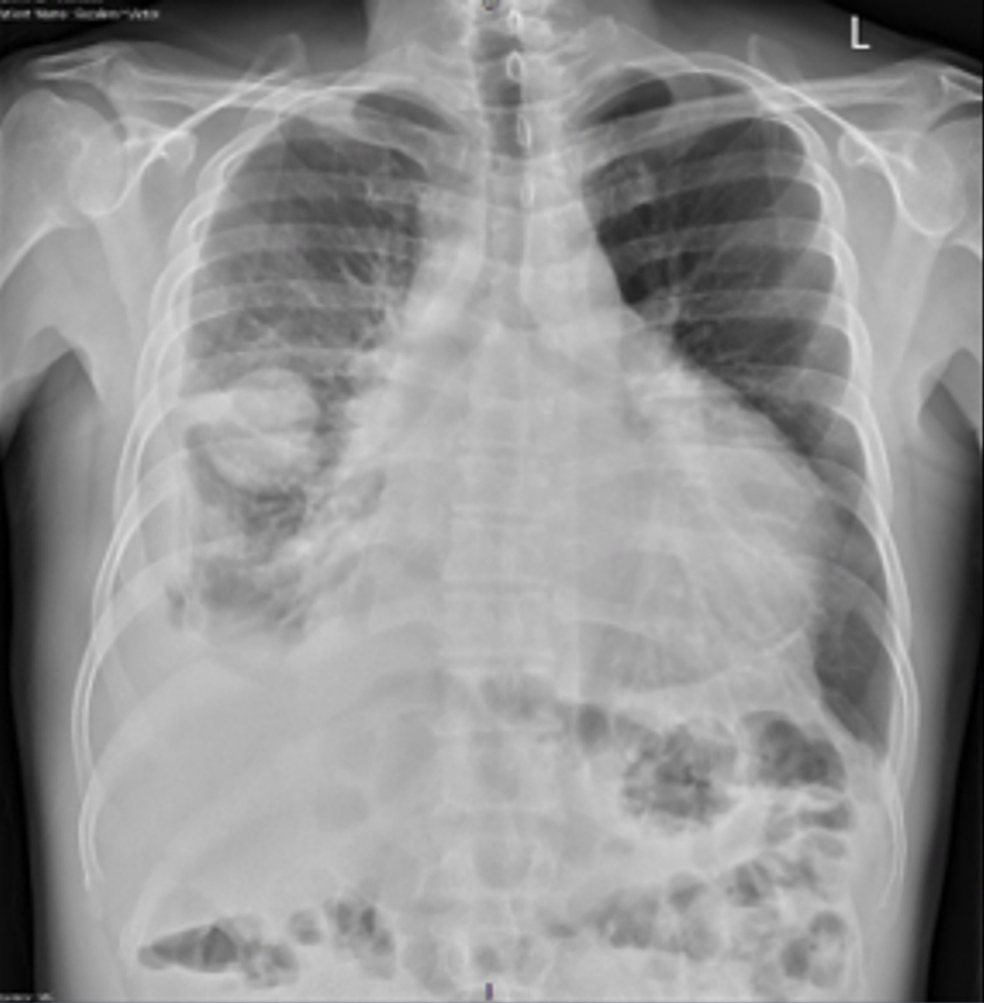
Chest X-ray of the patient The image shows mass projecting over the anterior mid lung in the region of the minor fissure, enlarged cardiac silhouette, and trace left pleural effusions

Laboratory data on admission were remarkable for mild hyponatremia of 133 mEq/L, chloride of 95 mEq/L, and Ca of 8.5 mg/dL. Complete blood count, rest of electrolytes, and liver enzymes were within normal ranges. Renal function tests were also normal, with a blood urea nitrogen (BUN) level of 17 mg/dL and a creatinine level of 1 mg/dL. Pro-brain natriuretic peptide (proBNP) was 341.2 pg/mL (normal range: 1-125 pg/mL). The patient had an erythrocyte sedimentation rate (ESR) of 10 mm/hr, C-reactive protein (CRP) of 2.42 mg/L, and his HIV antibody/antigen (AG/AB) screen was non-reactive. Chest X-ray showed an oval, 9.0 x 5.6 x 4.3-cm mass with an air-fluid level, projecting over the anterior mid lung in the region of the minor fissure, enlarged cardiac silhouette, and trace left pleural effusions (Figure 1).

**Figure 2:**
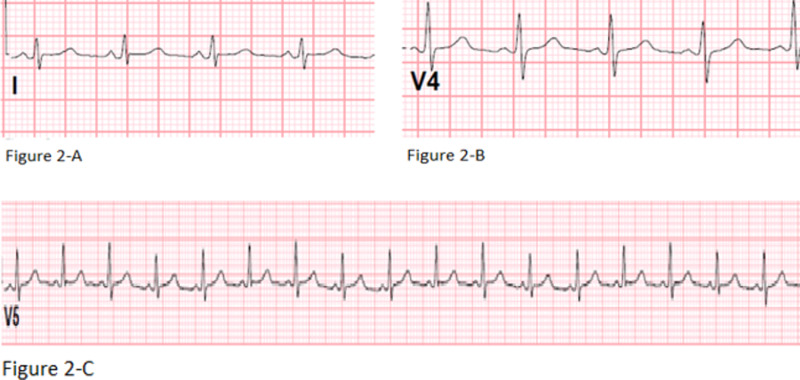
EKG evidence: low voltage QRS (2-A and 2-B) and electrical alternans (2-C) EKG: electrocardiogram

The figure below shows EKG evidence: low voltage QRS (Figures 2A, 2B) and electrical alternans (Figure 2C).

**Figure 3:**
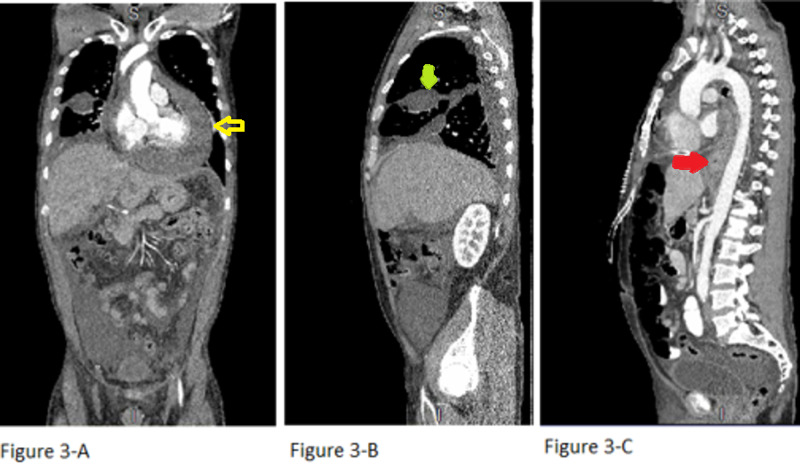
Abdomen and chest CT with contrast Large circumferential pericardial effusion measuring up to 4.3 cm, bilateral pleural effusions larger on the right with associated compressive and dependent atelectasis (3-A) with pleural fluid accumulation within the middle and lower fissure (3-A and 3-B), esophageal wall thickening (3-C), and mediastinal and hilar adenopathy CT: computed tomography

Abdomen and chest CT with contrast showed a large circumferential pericardial effusion measuring up to 4.3 cm, bilateral pleural effusions larger on the right with associated compressive and dependent atelectasis (Figure 3A) with pleural fluid accumulation within the middle and lower fissure (Figures 3A, 3B), esophageal wall thickening (Figure 3C), and mediastinal and hilar adenopathy.

Emergency bedside echocardiogram showed pericardial effusion with early right ventricular collapse. The patient underwent surgical drainage of pericardial fluid with a pericardial window.

**Figure 4:**
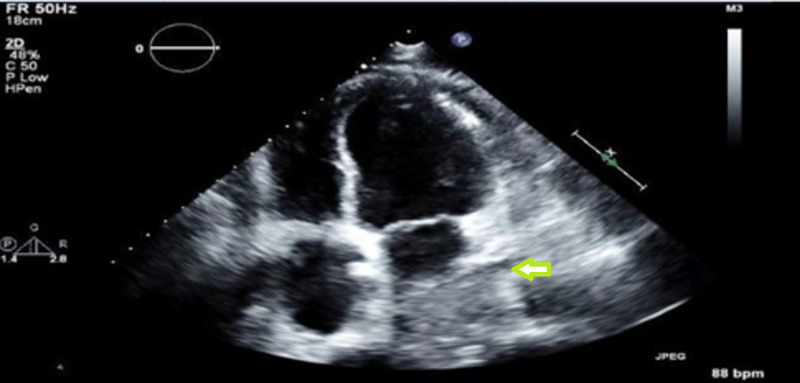
Echo after the surgical procedure showing a mass compressing the left atrium (arrow)

The pericardial fluid analysis revealed the following findings - cloudy appearance, total WBC of 55, RBC of 1,600, segmental neutrophils: 75%, lymphocyte: 25%, lactate dehydrogenase (LDH) of 492 U/L, protein of 2.9 g/dl, amylase of 9 U/L, and adenosine deaminase of 1.8 U/L. Serum LDH was 329 U/L and serum protein was 4.9 g/dL. Pericardial fluid cultures showed no growth. The echo after the surgical procedure showed a mass compressing the left atrium (Figure 4). Thyroid-stimulating hormone (TSH) was normal at 1.71 IU/mL. The following results were also observed - tick-borne Ab panel, serum: <1:64; Coxsackie A Ab: 1:400.

**Figure 5:**
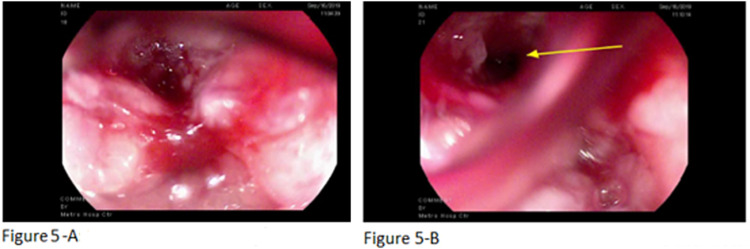
Esophageal tumor extending from the middle third to the GE junction of 31-43 cm (5-A), and an esophageal fistula (5-B) (arrow) GE: gastroesophageal

Pericardial biopsy and immunochemistry were positive for CK-20, and further analysis revealed adenocarcinoma of colorectal origin. Colonoscopy was performed, but only a mixed hyperplastic/adenomatous polyp was found. Due to the patient’s recurrent gastroesophageal reflux disease (GERD) symptoms and distal esophageal wall thickening on CT, he underwent esophagogastroduodenoscopy, which revealed the presence of likely esophageal malignant tumor extending from the middle third to the gastroesophageal junction of 31-43 cm (Figures 5A, 5B). Esophageal biopsy evidenced esophageal adenocarcinoma, moderately differentiated. Mucicarmine stain was focally positive.

## Discussion

The etiology for pericardial effusion can be divided into trauma, infections, malignancies, and connective tissue diseases. Malignant involvement of the pericardium is detected in 1-20% of cancer cases in autopsy studies [4]. In one study of 1,029 autopsy cases in which a malignant neoplasm was diagnosed, the authors found metastasis to the heart in 10.7% of the cases [5]. The same study identified lung as the most frequent primary site (36.4%), and adenocarcinoma was the most frequent cell type (36.4%) of neoplasms metastatic to the heart.

Large symptomatic pericardial effusions may present an unrecognized underlying malignancy in approximately one-fifth of the patients with a non-revealing basic workup [6]. In one study involving 173 patients who underwent pericardiocentesis during the study period, 58 patients (33%) were found with neoplastic pleural effusions, 45 of whom had a known malignant disease at the time of pericardiocentesis. The pericardial illness was found to be the initial presentation of an unrecognized underlying neoplastic disease in 13 patients (7.5% of all etiologies).

Malignant effusion tends to slowly stretch out the pericardial membranes, which accommodate the enlarging fluid volume without significant change. Still, once the stretch limit is reached, pressure starts to compress the cardiac chambers [7]. The most commonly seen clinical manifestations of metastatic heart diseases are dyspnea, cough, and pleural effusion rather than the direct effects of the tumor on the heart itself [8].

On the other hand, esophageal cancer is the eighth most common cancer and the sixth most common cause of death worldwide [9]. In a contemporary series, approximately 6-10% of the subjects were asymptomatic at the time of diagnosis [10]. Among patients with locally advanced esophageal cancer, progressive dysphagia, accompanied by weight loss, is the first symptom.

The survival rate in patients who present with pericardial effusion is meager. According to one of the prognosis studies of the survival curve, involving nine patients with large pericardial effusion secondary to metastatic disease, 100% died within five months after pericardiocentesis (mean survival: 2.4 ± 1.5 months) [11].

## Conclusions

Predominantly, dysphagia and weight loss are the first manifestations of esophageal cancer. Nonetheless, acute clinical presentations as cardiac tamponade may be the first sign of the disease in rare cases. Physicians need to consider esophageal cancer as one of the differential diagnoses for patients who present with cardiac tamponade once other causes are excluded, especially in the presence of risk factors for esophageal cancer.
